# Label-Free G-Quadruplex Aptamer Fluorescence Assay for Ochratoxin A Using a Thioflavin T Probe

**DOI:** 10.3390/toxins10050198

**Published:** 2018-05-12

**Authors:** Kefeng Wu, Changbei Ma, Han Zhao, Hailun He, Hanchun Chen

**Affiliations:** School of Life Sciences, Central South University, Changsha 410013, Hunan, China; kefeng@csu.edu.cn (K.W.); 172511001@csu.edu.cn (H.Z.); helenhe@csu.edu.cn (H.H.); chenhanchun@csu.edu.cn (H.C.)

**Keywords:** ochratoxin A, thioflavin T, G-quadruplex aptamer, fluorescence assay

## Abstract

Ochratoxin A (OTA) is one of the most common mycotoxins contaminating feed and foodstuffs. Therefore, a great deal of concern is associated with AFB1 toxicity. In this work, a fast and sensitive fluorescence aptamer biosensor has been proposed for the OTA assay. In the absence of OTA, the OTA aptamer can form a G-quadruplex structure with thioflavin T (ThT) dye, which results in increased fluorescence. After joining OTA, OTA aptamer combines with OTA and the G-quadruplex can be formed. Only faint fluorescence was finally observed when ThT weakly reacts with the quadruplex. Through this test method, the entire reaction and analysis process of OTA can be completed in 10 min. Under optimal experimental conditions (600 nM OTA-APT, 7 μM ThT, and 3 min incubation time), this proposed assay has a good limit of detection (LOD) of 0.4 ng/mL and shows a good linear relationship within the range of 1.2–200 ng/mL under the best experimental conditions. This method has a high specificity for OTA relative to Ochratoxin B (23%) and Aflatoxin B_1_ (13%). In addition, the quantitative determination of this method in real samples has been validated using a sample of red wine supplemented with a range of OTA concentrations (1.2 ng/mL, 12 ng/mL, and 40 ng/mL) with recoveries of 96.5% to 107%.

## 1. Introduction

Ochratoxin A (OTA) is a well-known mycotoxin produced by various Aspergillus and Penicillium strains and found in many foods such as cereals, coffee, cocoa, dried fruit, wine and grape juice, beer, spices, meat, and meat products [1,2,3,4,5,6]. OTA has a broad range of toxicological effects on animals and humans including neurotoxicity, immunotoxicity, teratogenicity, reproductive toxicity, and carcinogenicity [7,8,9,10]. OTA is easy to absorb and slowly decomposes. In the body, OTA metabolizes very slowly with a half-life of more than 30 days. Unlike other toxins, OTA is resistant to both low and high temperatures [11,12,13]. Humans absorb OTA mainly from moldy agricultural products and spoiled foods. Research reveals that this toxin can cause a wide range of health problems [14,15]. International and government agencies in many countries manage OTA content in food. Therefore, ensuring food safety and eliminating the potential risks posed by OTA is of great significance and it is necessary for developing an efficient and fast method for OTA monitoring.

The conventional methods for OTA analysis include Thin Layer Chromatography (TLC), High Performance Liquid Chromatography (HPLC), Gas Chromatography (GC), and Mass Spectrometry (MS) [16,17,18]. Although these methods have shown successful results for OTA detection, they involve expensive and laborious sample preparation steps. In order to overcome these problems, some recent testing methods have been developed. Chen et al. developed a simple and rapid biosensor for OTA based on a structure-switching signaling aptamer [19]. Lei et al. demonstrated a signal amplification strategy based on nuclease-aided target recycling for OTA detection [20]. Jo et al. presented the detection of OTA in coffee using chemiluminescence [21]. However, in these strategies, the aptamer needs to be labeled with fluorophores. 

Aptamers are single-stranded nucleic acids or peptide molecules exhibiting unique secondary structures, which can bind specifically to their targets. Aptamers can be used for the detection of small molecules, proteins, nucleic acids, cells, tissues, and organisms. Furthermore, aptamers perform better in synthesis, maintenance, and delivery, which demonstrate the ability of aptamers to be promising molecular receptors for bioanalytical applications [22,23,24,25,26]. Recently, a series of aptamer-based methods for detecting OTA has been reported. These assays mainly include fluorescence [27,28], electrochemical assays [29,30], colorimetric analysis [31,32,33], nanomaterials [34,35], and photoelectrochemical immunosensors [36]. Thioflavin T (ThT), which is a commercially available water-soluble fluorogenic dye, has been demonstrated to selectively bind to G-quadruplexes. This results in a significant enhancement of fluorescence. This finding has led to the development of G-quadruplex-based fluorescent sensors. However, the exploration of ThT is still at an early stage and has a great potential to be utilized in biochemical and biomedical applications. In this present study, we, for the first time, prepared a label-free fluorescence aptasensor for OTA determination based on ThT/G-quadruplex [37,38,39,40,41,42]. This proposed fluorescent assay is rapid, easy to operate, and can be applied to detect OTA in red wine.

## 2. Results and Discussion

### 2.1. Aptasensor Principles

**Scheme 1:**
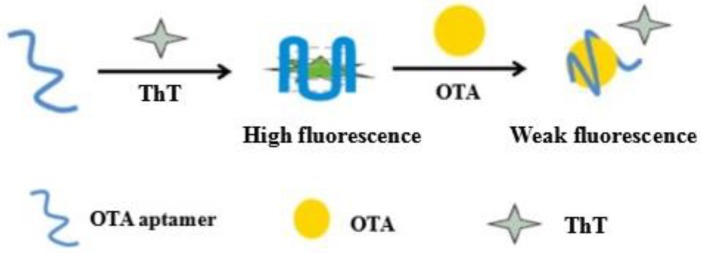
Schematic illustration of the fluorescent aptamer sensor for OTA. In the absence of OTA, OTA-Aptamer can form a G-quadruplex structure with thioflavin T (ThT), which results in enhanced fluorescence. After addition of OTA, OTA-Aptamer specifically binds to OTA and forms the G-quadruplex. After this quadruplex has a weak reaction with ThT, weak fluorescence is observed.

In this study, a fast fluorescence aptasensor for ochratoxin A determination was proposed and the design principle is illustrated in Scheme 1. The OTA-APT probe is the aptamer of OTA. In the absence of OTA, OTA-APT can form a ThT/G-quadruplex complex with ThT, which result in increased fluorescence. After joining OTA, the OTA aptamer combines with OTA and forms the antiparallel G-quadruplex. Due to a weak reaction between ThT and G-quadruplex, only faint fluorescence was observed. According to the change of a fluorescence signal from the detection system, we can easily detect OTA concentration.

### 2.2. Feasibility Test

**Figure 1:**
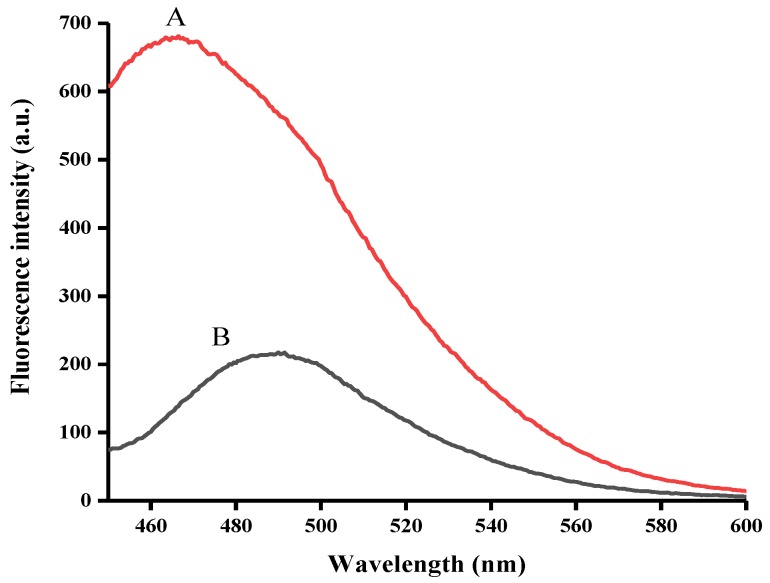
Fluorescence emission spectra in the (**A**) absence and (**B**) presence of OTA.

To demonstrate the feasibility of our method, two samples were prepared. Sample A contains the OTA-APT probe only. Sample B contains the OTA-APT probe and OTA. Figure 1 shows the fluorescence emission spectra of the OTA-APT probe both in the absence (curves A) and presence (curves B) of OTA. As demonstrated in Figure 1, the fluorescence emission intensity was quite low in the presence of OTA (curve B). This was expected since in the presence of OTA, the OTA aptamer combines with OTA and the antiparallel G-quadruplex structure can be formed. As shown in curve A, the OTA aptamer can form the G-quadruplex structure with ThT dye in the absence of OTA, which results in an increase in the fluorescent signal. This method established a simple but effective strategy for OTA concentration detection.

### 2.3. Optimization of the Aptameric Sensing Experiment

**Figure 2:**
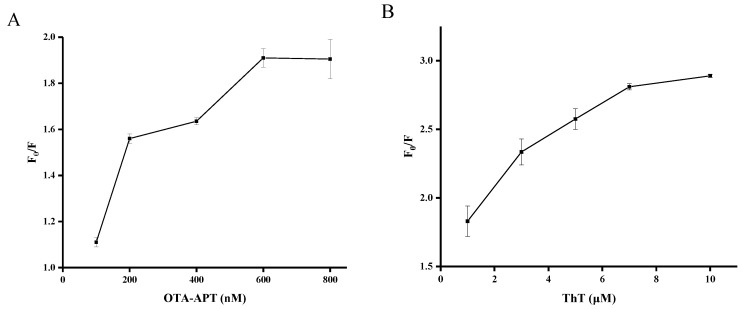

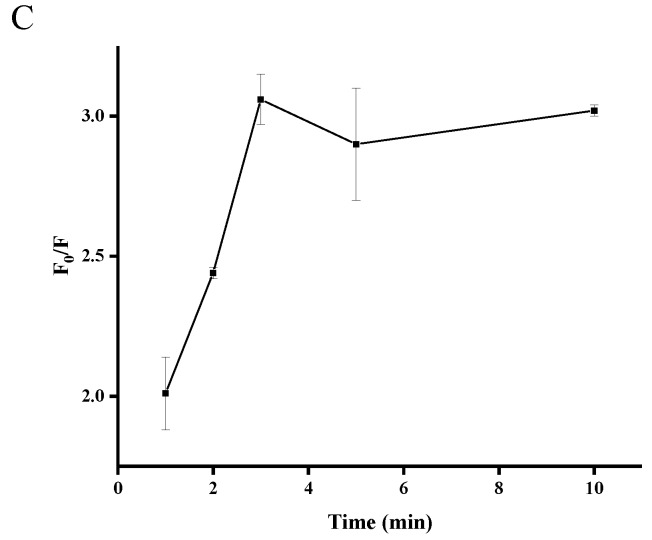
Optimization of OTA experimental conditions: (**A**) concentration of OTA-APT probe, (**B**) concentration of ThT, and (**C**) reaction time. F_0_ and F represent the fluorescence intensities in the absence and presence of OTA, respectively. Error bars are standard deviations of three experiments.

In order to obtain a desirable fluorescence response, experimental conditions were optimized including the concentration of OTA–APT, the concentration of ThT, and the reaction time. As shown in Figure 2A, the fluorescence signal enhancement was strongly dependent on the concentration of OTA-APT with the optimal concentration achieved at 600 nM. As shown in Figure 2B, the ratio of the increase in fluorescence intensity reached a plateau at 7 μM of ThT. Therefore, the optimized concentration of ThT was 7 μM in this detection method. As shown in Figure 2C, the incubation time of OTA and OTA-APT was optimized. The result showed that the ratio of increase in fluorescence intensity almost reached a plateau within three minutes. Therefore, we chose three minutes as the OTA incubation time.

### 2.4. Quantitative Detection of OTA

**Figure 3:**
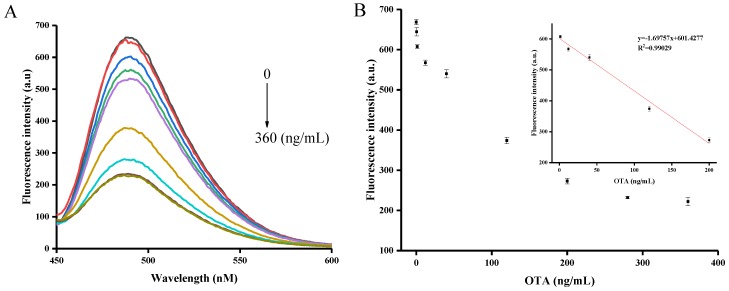
(**A**) Fluorescent signal response with different concentrations of OTA. OTA concentration decreased from bottom to top: 360, 280, 200, 120, 40, 12, 1.2, 0.4, and 0 ng/mL, (**B**) OTA assay showing the linear curve of the enhanced fluorescence intensity to concentration of OTA. The inset shows the linearity of the fluorescence intensity respect to OTA concentrations. Error bars are standard deviations of three experiments.

Under the optimized conditions, OTA was sensitively and quantitatively detected using the proposed aptasensor strategy. As depicted in Figure 3A, the fluorescence intensity of the system gradually decreased with increasing OTA concentrations in the range of 0–360 ng/mL. A calibration curve of fluorescence intensity as a function of concentration was plotted (see Figure 3B). This revealed that there was a sufficient linear correlation with a correlation coefficient of 0.99 between the fluorescence intensity and the OTA concentration in the range of 1.2–200 ng/mL (see Figure 3B). The limit of detection (LOD) was calculated to be 0.4 ng/mL according to the 3σ rule. As shown in Table 1, the OTA detection performance of our method was comparable to what was reported in most of the other studies. Therefore, it can be concluded that our assay technique may provide a simple and fast method for the sensitive quantification of OTA.

### 2.5. Selectivity of OTA Assay

**Figure 4:**
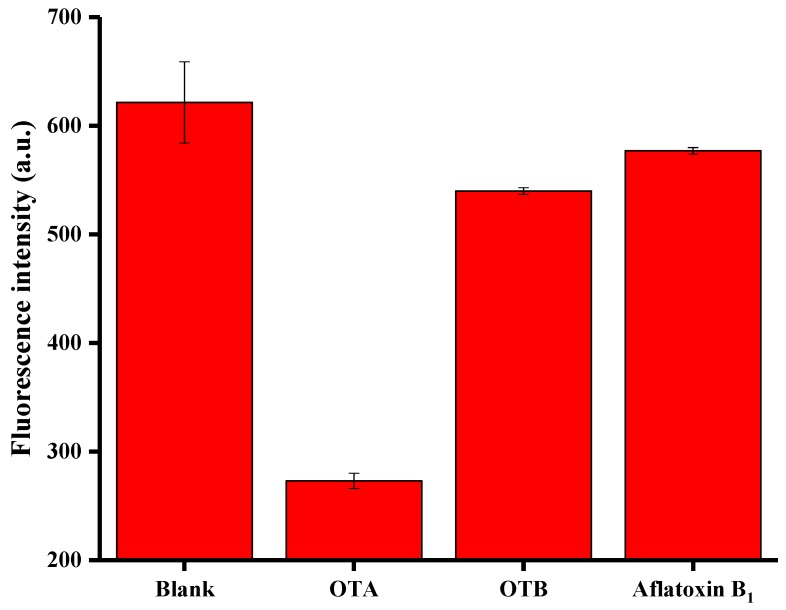
Selectivity of the proposed OTA (200 ng/mL) assay towards OTB and Aflatoxin B_1_ (360 ng/mL, respectively). Error bars are standard deviations of three experiments.

The specificity of the method was tested using OTB and AFB_1_. The experiments were performed using 200 ng/mL of OTA, 360 ng/mL of OTB, and 360 ng/mL of AFB_1_, respectively. It is clear from Figure 4 that only OTA can induce a remarkable signal attributed to the inherent specificity of the aptamer towards OTA. OTB and AFB_1_ resulted in only negligible fluorescent signal changes. The results demonstrate that the method has good selectivity.

### 2.6. Determination of OTA in Practical Samples

In order to test the feasibility and accuracy of the developed method, recovery tests were made in red wine samples. The aptasensor was used for determining the recoveries of three different concentrations of OTA (1.2 ng/mL, 12 ng/mL, and 40 ng/mL). The recoveries were found to vary in 96.5% to 107% (see Table 2), which confirmed the satisfactory accuracy of the assay for target OTA in a complicated sample matrix.

## 3. Conclusions

A fluorescence aptasensor was designed for fast detection of OTA based on ThT/G-quadruplex. Under the optimized conditions, this aptasensor exhibited a low detection limit of 0.4 ng/mL and a linear range of 1.2 ng/mL to 200 ng/mL for OTA. This detection method avoids the complicated synthesis and modification of sensing probes, which significantly lowered the detection cost. Furthermore, the aptasensor can be applied to the quantitative determination of OTA in real samples with satisfactory results. This strategy shows high sensitivity, good selectivity, and offers a wide range of applications in food safety-related fields. Moreover, this sensing strategy has the potential to be applied to other aptamer-based biochemical assays for the detection of small molecules in the fields of environmental monitoring and medical diagnostics.

## 4. Experimental

### 4.1. Materials and Measurements

Ochratoxin A (OTA) and ochratoxin B (OTB) were purchased from Pribolab Co., Ltd. (Qingdao, China). Aflatoxin B_1_ (AFB_1_) was purchased from Yuanye Co., Ltd. (Shanghai, China). The DNA probe (OTA-APT) was purchased from Sangon Biotechnology Co., Ltd. (Shanghai, China). Tris [Tris-(hydroxy-methyl)aminomethane], hydrochloric acid (HCl), potassium chloride (KCl), sodium chloride (NaCl), and calcium chloride (CaCl_2_) were purchased from Sinopharm Chemical Reagent Co., Ltd. (Shanghai, China). Thioflavin T (ThT) was purchased from Sigma-Aldrich (St. Louis, MO, USA). All other reagents were of analytical reagent grade and were used as received. Ultra-pure water (18.2 MΩ·cm^−1^) was used in all experiments. The DNA probe was dissolved in the Tris-EDTA buffer and stored at −20 °C until further use. OTA-APT sequences were marked by 5′-GATCGGGTGTGGGTGGCGTAAAGGGAGCATCGGACA-3′. The fluorescence spectra were obtained using a Hitachi F-2700 fluorescence spectrophotometer (Hitachi Ltd., Tokyo, Japan). The samples placed in quartz cuvettes were excited at a wavelength of 425 nm and all emission spectra were collected at wavelengths of 450–600 nm at room temperature.

### 4.2. Optimization of Experimental Conditions

In order to obtain the best experimental results, the relevant experimental conditions were optimized one by one including the concentrations of OTA-APT, the concentrations of ThT, and the reaction time. The concentration range of OTA-APT was 100 nM, 200 nM, 400 nM, 600 nM, and 800 nM. The ThT concentration was 1 μM, 3 μM, 5 μM, 7 μM, and 10 μM. The reaction time was 1 min, 2 min, 3 min, 5 min, and 10 min.

### 4.3. Aptamer Biosensor for Detecting OTA

For a typical OTA fluorescence aptasensor experiment, the OTA buffer (containing 10 mM Tris-HCl, 120 mM NaCl, 20 mM CaCl_2_, 5 mM KCl, pH = 8.5), 600 nM OTA-APT and different concentrations of OTA were incubated at room temperature for 3 min. Afterward, 7 μM ThT was added to the reaction solution. After 5 min, fluorescence intensity was measured with excitation at 425 nm and the emission spectra were collected in the range of 450–600 nm. All reactions were conducted at a final reaction volume of 100 μL for three times.

### 4.4. Selectivity Assay

In order to analyze the specificity of this experiment for OTA, we chose OTB and AFB_1_ as controls. At first, OTA buffer, 600 nM OTA-APT and 200 ng/mL OTA, and 360 ng/mL OTB and 360 ng/mL AFB_1_ were incubated at room temperature for 3 min. Following this, 7 μM ThT was added. After 5 min, we obtain fluorescence intensity measurements.

### 4.5. Determination of OTA in Practical Samples

In order to explore the feasibility of this method in real samples, red wine was used to test the feasibility. This was completed in order to reduce the influence of the color and residue of red wine on the experimental results. Red wine samples were filtrated first to remove the wine sediment and then diluted 20-fold for the subsequent analysis of OTA. Three different concentrations of OTA (1.2 ng/mL, 12 ng/mL, and 40 ng/mL) were spiked into the red wine samples to obtain standard samples. The subsequent manipulation was conducted according to the reported strategy.

## Figures and Tables

**Table 1:** Comparison with the current reported methods for OTA Determination.

Method	Detection Limit (ng/mL)	Linear Range (ng/mL)	Ref
Gold nanoparticle	8	8–250	[32]
Gold nanorod	0.22	0.5–22	[35]
Carbon nanotube	4	4–80	[20]
Double label	0.8	1–100	[19]
SYBR Green I	4	4–40	[27]
CRET	0.22	0.1–100	[21]
Label-free	0.4	1.2–200	This work

**Table 2:** Application of aptasensor for OTA determination in red wine.

Sample Number	Added (ng/mL)	Detected (ng/mL)	Recovery (%)
1	1.2	1.28 ± 0.11	107
2	12	13.4 ± 0.25	111.6
3	40	38.6 ± 0.53	96.5
